# Evaluation of a supported education and employment program for adolescents and young adults with mental health problems: A study protocol of the StAB project

**DOI:** 10.1371/journal.pone.0271803

**Published:** 2022-07-29

**Authors:** Ina Schniedermann, Lorenz B. Dehn, Sabrina Micheel, Thomas Beblo, Martin Driessen, for the StAB project group

**Affiliations:** 1 Department of Psychiatry and Psychotherapy, University Hospital OWL of Bielefeld University, Bielefeld, Germany; 2 Department of Psychology, Bielefeld University, Bielefeld, Germany; Public Library of Science, UNITED STATES

## Abstract

The majority of mental illnesses begins in childhood, adolescence and young adulthood before the age of 25. The transition from adolescence to adulthood is a particularly vulnerable time for adolescents with mental illness, affecting psychosocial functioning and participation in work life. Therefore, they need—in contrast to classic standard vocational interventions—a long-term, holistic and individually oriented vocational rehabilitation program. With the innovative model project "Start in education and employment (StAB)”, adolescents and young adults with mental illnesses are to be supported with regard to their vocational perspectives and participation by a new type of individualized, holistic, long-term job coaching. It follows the Individual Placement and Support (IPS) concept, as a manualized form of the Supported Employment Approach and is based on the "first place–then train" principle. In order to evaluate the effectiveness and feasibility of the StAB program, a double-centre prospective single arm evaluation study in a mixed-methods design will be conducted. The focus is on quantitative research analysing pre-post-effects of the StAB intervention in a two-year observational study. Young people between 15 and 25 years with a psychiatric diagnosis who are currently in receipt of means-tested benefits or are entitled to them will be recruited. The study will take place in two major cities in the north-western part of Germany, Bielefeld and Dortmund. We expect to contribute to gain more empirical data about the implementation of Supported Employment and Education to severely mentally ill adolescents and young adults in German settings. Moreover, these results may also provide the scientific foundation for future measures focusing the improvement of vocational rehabilitation for young people with mental illness.

The study was registered in the German Clinical Trials Register (DRKS00027576) on March 10, 2022.

## Introduction

The majority of mental health illnesses have their roots early in life. In fact, it has been found that 75% of all psychiatric disorders begin before the age of 25 [1–3]. This period between the ages of 15 and 25 years is a sensitive phase in everyone’s life, in significant neurobiological, cognitive, social and legal changes are imminent and different developmental milestones need to be achieved [4]. However, for young people with mental illness, the transition from adolescence to adulthood is particularly challenging because of diminished social skills and coping strategies compared to their not affected (healthy) peers [5] and they have to face great difficulties in gaining access to mental health services [6]. Moreover, mental illnesses are often accompanied by serious impairments of psychosocial functioning, which in turn makes it difficult to participate in various areas of life. For example, mental health problems are often associated with negative effects on the educational and employment situation [7]. As a result, limited participation in working life is accompanied not only by economical losses, but also by the absence of psychosocial benefits, such as access to social networks or a sense of purpose and meaning. As Lloyd and Waghorn (2007) have pointed out, work has an important role in recovery and rehabilitation, because “*having a reason to get out of bed and something meaningful to do during the day is essential for the well-being of people with mental illness*” [8] (p. 127). However, adolescents with psychiatric disabilities often face significant barriers to employment, e.g. lack of work experience, transportation problems, lack of clear responsibility for promoting vocational development or psychiatric symptomatology not being well controlled [8, 9]. On the other hand, research has shown that access to employment support programs for young people with mental illness may be associated with higher vocational success rates and improved long-term employment outcomes [10].

Whereas traditional vocational rehabilitation programs train people in certain required skills before (re)placing them in jobs, the supported employment (SE) approach foregoes any extended preparation but concentrates on placing participants in the labour market as soon as possible [11]. Systematic reviews revealed that the Individual Placement and Support (IPS) program, as a manualized form of SE, is highly effective in comparison to other regular “first train–then place” rehabilitation programs [12, 13]. The central features of IPS are: eligibility based on client choice, focus on competitive employment, integration of mental health and employment services, attention to client preferences, work incentives planning, rapid job search, systematic job development, and individualized job supports [14]. Although IPS has become a well-established practice model and is widely applied to help adults with severe mental illnesses [15, 16], evidence on its effectiveness for adolescents and young people is still sparse [17–19] especially in Germany [20]. The German social and health care system, similarly to other European countries [21], is very fragmented and characterized by different intersections, leading to discontinuities and gaps in the psychosocial care of people with mental health difficulties [22]. Therefore, a long-term, coordinated, holistic and individualised support for education and employment is of high relevance during the vulnerable transition from adolescence to adulthood. With our study presented here, we want to contribute to the research and dissemination of an IPS oriented intervention for young people with mental health difficulties between the ages of 15 and 25. The study protocol for the evaluation of this innovative support program is described throughout the present paper. This study protocol is the first version (16.05.2022).

## Materials and methods

### Objectives

The main objective of this study is to evaluate the effectiveness and feasibility of the StAB project (German: Start in Ausbildung und Beruf, [= Start in education and employment]), a supported employment and education intervention for mentally burdened adolescents and young adults. Based on the IPS approach, StAB aims at facilitating entry or re-integration to the general labor market or regular vocational training.

We assume that the intervention will result in an increased psychosocial participation and functioning of the participants (primary outcomes). At the same time, barriers and inhibiting living conditions are expected to decrease, so that quality of life, self-efficacy and other dimensions of the participants will improve. In terms of occupational parameters, we expect at least 40% of the participants to have completed education or vocational training or a specific preparatory internship, or to have returned to work or are employed at the primary labour market within two years after program enrolment. In addition, the effects of the intervention will be analyzed regarding further outcome variables, e.g. self-esteem, psychopathological symptoms or need for inpatient treatment.

The StAB project also aims to structurally change the local health and social care system. For this purpose, the implementation of a cross-sector competence network with relevant stakeholders and key informants from the fields of pediatric and adolescent medicine, child and youth work, education, vocational rehabilitation, and psychosocial support is planned.

### Study design and setting

The StAB supported employment and education program is realized by a mixed-methods design. Its main focus is a naturalistic, double-center prospective single arm evaluation study using a pre-post repeated measure design. Given the vulnerable target population of young people with different mental health problems, this study will also examine the feasibility of program implementation and does not include a control group. The establishment of a competence network will be supported by qualitative and quantitative surveys in order to identify the needs of stakeholders in the field of vocational integration and rehabilitation for young people with mental health problems. The study will take place in Dortmund and Bielefeld, two major cities in the north-western part of Germany. Recruitment is scheduled to begin during May 2022. The trial was registered at the German Clinical Trials Register on 10/03/2022 (DRKS00027576).

### Participants

To be eligible to the study, potential participants have to fulfil the following inclusion criteria: A) age 15–25 years, B) a psychiatric diagnosis (either currently or in the last 12 months) according to International Statistical Classification of Diseases and Related Health Problems (ICD-10) criteria, excluding acute intoxication and intellectual disabilities, C) receiving or currently applying for means-tested benefits (German: Arbeitslosengeld II), and D) sufficient written and spoken German language skills. All participants are required to read the study information and sign the consent form. For minors between 15 and 17 years of age, written informed parental consent is required.

### Sample size

With regard to the central outcome parameters, an improvement in the questionnaire results (e.g. psychosocial participation and functioning, quality of life, self-efficacy) between the initial and final assessment is expected over the two-year StAB intervention period. Thus, considering A) a paired t-test for dependent samples as the statistical analysis method, B) a small to medium effect size (d = 0.4), C) an α-(error) level of 0.05, and D) a β-level of 0.2 (or a power of 0.8), the a priori case number estimation using the programme G*Power 3.1 [23] resulted in a required sample size of n = 52 persons. Assuming a dropout rate of at least 30% during the 24 months period after recruitment, at least 75 people should be included into the study.

### Recruitment and study procedure

Participants will be recruited through local psychiatric clinics and the responsible job centers in both study sites. Involved staff members will be comprehensively informed about the project, e.g. during departmental or ward meetings, and receive written study information (project overview, inclusion criteria, access flow). When staff members have screened and identified patients matching the inclusion criteria, these potential participants will be provided with initial study information and then will get in contact with an IPS coach in order to receive more detailed oral and written information. Potential study participants will be given sufficient time to ask questions, and they also receive a detailed information sheet on the goals, nature, and implications of the study project as well as on data protection regulations. After the interested person has confirmed participation following an individual consideration and decision-making phase, and signed the informed consent, a study entry assessment appointment will be scheduled and the IPS coaching intervention (see next section) will start. As it is described in the study information, participants can leave the program at any time without any disadvantages arising for them. Recruitment is scheduled to begin in May 2022.

### Intervention

IPS coaching as the manualized, evidenced based form of Supported Employment (SE) for people with mental illness focusses on an individualized, rapid search for competitive employment according to the client’s needs and preferences [24]. In addition to the emphasis on an individualized job support, the integration of accompanying mental health and employment services also plays a major role. The Supported Education Approach (SEA) can be considered equivalent to SE, but focusses more on the career start. This means, Supported Education aims at enabling the (mentally burdened) young individuals to (a) set and achieve an educational goal (e.g., training certificate or degree), (b) improve educational competencies (literacy, study skills, time management), (c) navigate the educational environment (e.g., applications, financial assistance), and (d) improve attitude and motivation toward completing educational goals [17]. An overarching goal is to support the transition to sustainable employment or training relationship [17].

As both support approaches take into account the interrelationships of health stabilization, coping with everyday life, social environment as well as professional development, they represent holistic concepts of coaching [25]. In line with this, the StAB intervention will also conceptualize the IPS coaching intervention as a kind of “lifeworld coaching”, including a wide variety of topics, depending on the support desired or needed by the participant. Additionally, a peer-counselling component will be implemented as part of the intervention program. In general, the StAB programs concept of supported education and employment is intended to overcome various individual challenges, such as 1) solving individual problems in different areas of life, 2) exploring one’s own strengths and interests and developing a professional goal, 3) providing information needed by the participants for professional orientation and for taking up training or employment, 4) planning an application as well as supporting the search for a suitable job / training position, 5) preparing a good start at the apprenticeship or workplace, 6) discussing the arrangements of the participant’s training or job in the educational institutions and companies.

Besides the fragmented German social system (see above), the StAB program is not (yet) feasible under regular conditions of means-tested benefits in Germany, since vocational placement efforts are put on hold while the initial focus is on clarification of the (mental) health situation and the utilization of psychosocial treatment options, stabilization of the mental health situation and rehabilitation of working ability [26].

The participation in the StAB program offers improved opportunities to a sustainable entry into working life, it is voluntary and free of charge. The StAB program consists in assigning an IPS coach to each participant in order to provide individualised support for integration into labour market for the young adults and adolescents with mental illness. Thereby, a bridge to (re-)entry into training and work is to be built in parallel to the regular psychiatric treatment. The IPS coaching takes place exclusively in individual contacts. These contacts are coordinated in terms of timing and content according to the respective needs and the therapeutic treatment of the participants, focussing on the current situation by addressing stagnations in development, setbacks or crises. Moreover, as IPS coaching aims at strengthening the self-help potentials of the young people [27], the participants should play an active role in working out and achieving their goals. Therefore, IPS coaches in the StAB program do not just give advice but work out options together in order to have a positive effect on the participants’ own responsibility. This is an important aspect in order to evoke acceptance of the current health conditions and to focus on the resources of the adolescents and young adults. For this purpose, the coach is able to use various interventional approaches, aiming at sensitising the young people to perceive their own resources, to recognize critical situations for them and to reflect on their “usual” behaviour in order to try out new options for behaviour and action in a next step. If there are needs for support in the areas of housing, finances, authorities and agencies etc., the coach establishes contact with the relevant counselling centres, if this has not already been done by the responsible integration specialist at the job centre. The IPS coaches accompany the professional process with the psychiatric and social-pedagogical specialists and create helpful support and network structures. The integration planning takes place in coordination with the regular psychosocial treatment and integration system. Thus, the appropriate services from the regular social care system should also be considered as soon as vocational perspectives and the necessary implementation steps have been worked out. Treatment as usual introducing people to the labour market in Germany involves for example group-delivered vocational preparation or trial employment. In addition to the coaching process, the StAB program will be able to provide an “individual budget” to its participants, which can be used for individual rehabilitation or participation planning, if necessary. The individual budget is €50 for each participant per month, whereby the amount of the budget can vary and the total sum should be usable as a total budget.

In order to ensure a uniform standard of IPS coaching, the StAB coaching team will receive a modular training at the beginning and during the course of the project, including for instance motivational interviewing, basics of social law, and in particular the impact of mental illness on learning and working as well as the role of the coach with regard to participants and employer / training company. Although according to the IPS principles coaching is not time limited, there is a maximum duration of 30 months in our StAB program (due to the model character of this third-party funded study). However, during this period, participants receive comprehensive long-term support, even though they are already in employment or training.

Another component of the project is the establishment of a competence network with relevant actors from the fields of child and youth work, school, training, vocational participation and work as well as the psychosocial and psychiatric system. Through joint meetings and accompanying mixed-methods surveys of the competence network, needs for improvement will be identified, future strategies for the health and social care system will be developed, and joint cooperation agreements will have been ratified at the end.

### Assessments

The baseline assessment is intended to acquire data on different participant characteristics by means of a study-specific questionnaire, e.g. demographics, educational status, employment history, work situation, utilization of (mental) health services, and expectations towards study participation. Furthermore, several standardized questionnaires (see below) will be handed out to participants for self-completion, but they can receive support or clarification from the research team member at any time if they want to. In addition, a brief screening test of cognitive functioning will also be performed. The whole assessment procedure is expected to take approximately 75 to 90 minutes. It will be carried out by experienced, trained and supervised research team members that can support participants that have questions regarding the questionnaires. In addition to the baseline assessment (t0), there will be a short interim assessment after one year (t1) and a final assessment at the end of the intervention after a maximum of two and a half years (t2). The t2 assessment is announced early and repeatedly, and its implementation is closely coordinated with participants to achieve high participation rates, even among potential dropouts.

Moreover, in addition to the study assessments we plan to include standardized process data from the IPS coaches to obtain information on frequency, amount, and type of support across the course of the StAB program. An overview of all variables, instruments and outcome measures utilized in the study is given in Table 1.

**Table 1:** Diagnostic instruments and assessment schedule (the full names of the instruments are given in the text, see Measures).

		STUDY PERIOD		
		T0 (baseline assessment)	T1 (short interim assessment)	T2 (final assessment)
**TIMEPOINT** (months)		0	12	24
**ENROLMENT**				
Eligibility screen		✓		
Informed consent		✓		
**INTERVENTIONS**				
IPS-Coaching				
**ASSESSMENTS**				
Psychosocial participation and functioning	IMET	✓		✓
	WHODAS 2.0	✓	✓	✓
Mental health status	PHQ-9	✓	✓	✓
	SCL-K-9	✓	✓	✓
	L-1	✓	✓	✓
	SCIP	✓		✓
	AMS-R	✓	✓	✓
Self-esteem and self-efficacy	G-SISE	✓	✓	✓
	ASKU	✓	✓	✓
Psychosocial resources	Social contacts (LK-18)	✓	✓	✓
	BRS	✓		✓
	HLS-EU-Q16-GER	✓		✓
Study-specific questionnaires	Demographics, Professional biography, Work ability, Satisfaction with medical care, Work and employment prospects under corona conditions	✓	(✓) partially	✓

#### Measures

Table 1 shows the questionnaires as well as their measurement time point within the study.

*Psychosocial participation and functioning*. Developed by the WHO, the **WHODAS 2.0** (*World Health Organization Disability Assessment Schedule 2*.*0*) is an assessment instrument to measure health, functional impairment and disability across diseases, countries, and cultures in accordance with the International Classification of Functioning, Disability and Health (ICF) [28]. In our study we use the 12-item self-report version, that has been found to explain 81% of the variance of the most widespread 36-item version [28]. The 12-item WHODAS 2.0 presents high internal consistency (up to 0.96), reliability, sensivity to change as well as overall good correlations with other measures of disability [29].

The **IMET** (*Index zur Messung von Einschränkungen der Teilhabe*, German for: Index for the Assessment of Health Impairments [30]) was developed to assess participation and involvement for patients with different (chronic) diseases as described by the ICF. The extent of impairment in nine areas of everyday activities (e.g. family/household obligations, recreational and leisure activities) have to be indicated on an 11point scale from 0 = no impairment to 10 = no more activity possible. Cronbach’s alpha was .90 - .91. Convergent validity is supported by moderate to high correlations of the IMET total score with earning capacity and performance measures. Furthermore, normative data from a population-based survey allows classification of participation impairment [31].

*Mental health status*. For assessing the burden of psychopathology, the *9-item short version of the Symptom Checklist* (**SCL-K-9**) is used [32]. This short scale measures the subjectively perceived impairment by physical and psychological symptoms within a period of seven days. The SCL-K-9 exhibits satisfactory psychometric properties and its summed up total score is highly correlated with that of the original 90 items questionnaire (r = .93) [32], thus representing a Global Severity Index (GSI, [32]).

In order to assess the presence and severity of depressive symptoms, we chose the **PHQ-9**, which is based on the 9-item depression module from the *Patient Health Questionnaire* [33]. The PHQ-9 comprises the diagnostic criteria of major depression according to the *Diagnostic and Statistical Manual of Mental Disorders*, but also serves as a severity measure since each of the 9 items can be scored from 0 (not at all) to 3 (nearly every day). As a well-established depression screener, the PHQ-9 has been found to have good psychometric characteristics as well as diagnostic validity and can be used to detect depression outcome and changes over time (e.g. [34]).

General life satisfaction will be measured using *The General Life Satisfaction Short Scale* (**L-1**) [35, 36]. This scale only consists of one item asking “All things considered, how satisfied are you with your life these days?” with 11 response categories ranging from “not satisfied at all” (0) to “completely satisfied” (10). As part of its development, the L-1 was evaluated with regard to its methodological quality in population samples from Germany and England [35, 36]. Although psychometric differences may appear when compared to multi-item equivalents [37], recent research findings support the use of single-item life satisfaction scales [38].

The *Screen for Cognitive Impairment in Psychiatry* (**SCIP**) is a brief and easy to administer screening tool for cognitive performance specifically developed for psychiatric populations [39]. It includes five subtests for assessing a) immediate and b) delayed verbal learning, c) working memory, d) verbal fluency, and d) processing speed. Moreover, the SCIP is available in different parallel forms to facilitate a prospective assessment of the cognitive status. The applicability and methodological criteria of the SCIP have been verified in different countries [40–42] and recent study results with the German version [43] showed that it is a valid and reliable cognitive screening tool.

*Self-esteem and self-efficacy*. The global self-esteem level will be measured using the *German version of the Single-Item Self-Esteem Scale* (**G-SISE**) [44]. The G-SISE asked participants to respond to the statement “Please indicate to what extent the following statement applies to you: I have high self-esteem.” on a 5-point Likert scale (1 = “not at all true of me” to 5 = “very true of me”). Studies have not only identified convergent correlations of around r = .75 between the SISE and the well-known 10-item Rosenberg Self-Esteem scale, but also supported the reliability and validity of the ultrashort scale [44, 45].

The GSE-3 (*General Self-Efficacy Short Scale–3*) consists of three items and is used to economically measure subjective competence expectations to be able to deal with difficulties and obstacles in daily life [46]. The short scale has a unidimensional structure, reliability varies between .73 and .92, and scalar measurement invariance holds across different European countries [44, 45]. In the present study, the original German-language scale of the GSE-3, called “Allgemeine Selbstwirksamkeits Kurzskala” (**ASKU**), will be applied [47].

We use the *revised 10-item version of the Achievement Motives Scale* (**AMS-R**) to assess hope of success and fear of failure [48]. The AMS-R was adjusted with regard to the factorial structure and provides adequate reliability as well as criterion-related validity with respect to typical achievement-related behaviour. Research studies have indicated that motivational variables seem to relate to psychosocial and vocational functioning in psychiatric patients [49, 50].

*Psychosocial resources*. Measuring the ability to recover from stress, the German adaptation of the *Brief Resilience Scale* (**BRS**) [51] consists of six items (e.g. “I tend to bounce back quickly after hard times”), that have to be rated on a five-point Likert scale ranging from 1 = strongly disagree to 5 = strongly agree. Results from studies with large (representative) samples yielded evidence for the reliability and validity of the German BRS and also provided population-based norms [51, 52].

The German version of the Rasch scaled short form of the *European Health Literacy Questionnaire* with 16 items (**HLS-EU-Q16**) captures different dimensions of (general) health literacy skills (finding, understanding, judging and applying health information) in the areas of disease prevention, health promotion and health care [53]. Respondents are asked to rate on a 4-point scale how easy they think each health literacy task or activity is (very easy—very difficult). For both the German (e.g. Cronbach’s α = 0.90) and further European versions, the HLS-EU-Q16 showed good results for internal consistency and other psychometric features [54, 55]. Following a recent approach [56], we additionally included another item from the original long form of the HLS-EU-Q47 [57] to compose a brief four-item Mental Health Literacy subscale.

In order to capture the involvement in social network structures with supportive relationships and close contacts, the “**social contacts**” subscale from the German Life Skills questionnaire (Lebenskompetenz-Fragebogen, LK-18) [42] will be used. It comprises three items (e.g. “I experience interpersonal support in life”) to be answered on a 5-point-Likert Scale. The subscale’s internal consistency was Cronbach’s α = .75.

*Further measures and questions*. As we want to assess participants’ **subjective work ability**, we therefore use several single items from different assessment tools (1) Würzburger Screening [58], 2) Short Screening Instrument of Need for Occupation Related Treatment in Medical Rehabilitation (SIBAR) [59], 3) Indicators of rehabilitation status, version 3 (IRES-3) [60].

In addition to the aforementioned measurement instruments, we will collect data on **various participant characteristics** across the study period using study-specific and self-constructed questionnaires. Here, the main focus is on professional and educational development, utilization of psychosocial support services as well as participation in and satisfaction with the program. Moreover, we also plan to include standardized process data from the IPS coaching team to obtain information on frequency, amount, and type of support across the course of the program. Additionally, the subjectively perceived impact of the corona pandemic on the education and employment situation of the participants will be obtained. For this purpose, questions from a recent short survey on vocational perspectives for people with mental illness under corona conditions will be used [61].

#### Data analyses

Before data analysis, the data set will be inspected with regard to inconsistencies and missing values. Missing data will be addressed by applying linear mixed models, if appropriate. We will perform a dropout analysis to check for differences between program completers and non-completers with regard to main demographic, psychosocial, and work-related variables. Besides descriptive baseline data analysis, we will primarily analyse changes in the main outcome measures from baseline to final assessment (after up to a maximum of 2.5 years). Theses analyses on the study outcome measures will be performed as intention-to-treat, supplemented by a “per-protocol”-analysis including all individuals who have completed the StAB program without dropping out. Of course, a repeated-measures approach is used if indicated. Program effects on outcome measures will be explored by taking into account relevant covariates, like key baseline variables, changes in some measures within the first year (see above) or program specific variations, e.g. number and total duration of coaching sessions. Program feasibility and acceptance among the participating adolescents and young adults with mental health difficulties will be evaluated using process documentation data as well as subjective ratings in the final assessment.

The statistical analysis of the data will be done using the software package IBM SPSS Statistics Version 25 and the level of statistical significance will be set to p < .05.

#### Research ethics and standards

The study will be performed according to the “Guidelines for Safeguarding Good Research Practice” by German Research Foundation (DFG) [62], the Declaration of Helsinki [63] and in compliance with the relevant dataprotection regulations. The trial was approved by the Ethics Committee of the Medical Association of Westphalia-Lippe and the University of Münster on 02.03.2022 (2022-037-f-S), which had no ethical or legal concerns about conducting the study. Moreover, the present study was registered at the German Clinical Trials Register (DRKS00027576) and will be updated there in case of modifications and adjustments.

#### Ethics approval and consent to participate

Ethical approval for this study is granted by the Ethics Committee of the Medical Association of Westphalia-Lippe and the Westphalian Wilhelms University Münster (2022-037-f-S). Before participating in the study, all participants will read an information statement and give informed consent. For participants who have not yet reached legal age, an informed consent from a legal guardian is needed. Any modifications of the trial protocol will be communicated to the Ethics Committee.

## Discussion

International research has clearly highlighted the importance of work for the self-development, social engagement and recovery of young adults with mental health problems [8, 64–66]. However, many adolescents and young adults experience barriers along their educational and work pathways that lead not only to unsteady and inconsistent employment trajectories but also unfavourable health and social outcomes [10, 67, 68]. Long-term studies have demonstrated that psychiatric disorders between the ages of 18 and 25 were associated with decreasing likelihood of paid employment, lower full-time employment; fewer hours worked per week, and increased rates of welfare dependence at age 30 [69, 70]. Moreover, young people with (severe) mental disorders are less likely than any other disability group to be included in, or to benefit from, regular employment programmes [71]. The very latest "Third Participation Report of the Federal Government on the Living Conditions of People with Impairments" also shows that in Germany, people with severe mental or psychological problems are particularly often affected by unemployment and severe problems with participation in working life [72].

Performing the StAB program we intend to take up the call for a wider dissemination of individualized supported education and employment for young individuals with mental health problems [67, 71]. The StAB program includes low-threshold inclusion criteria for adolescents and young adults with a wide range of psychiatric diagnoses, offers long-term coaching support, accompanying school attendance, training or the start of an employment; operates in collaboration and coordination with the regular support system; features the option for an additional flexible “individual budget” for participant-related integration service, and additionally aims to establish sustainable network structures with relevant actors and institutions from the social and health care system.

Nevertheless, the implementation and evaluation of the presented study is also associated with some challenges and limitations. For instance, we will face possible challenges with regard to the recruitment of the targeted group of people. Emerging adults with mental illnesses already face many difficulties to cope with a particularly vulnerable phase of life. It has been shown that they may not actively seek professional help themselves (see [73]) due to stigmatization and shame as well as a preference for self-reliance [74] and that they tend to disengage from ongoing intervention services by virtue of adverse vocational trends (e.g. [75]). Thus, accessibility to the program and reachability to these young people across the study period may be potentially difficult. We therefore expect a 30% dropout rate, in line with other IPS studies [76] and even slightly higher than comparable vocational programs for mentally burdened young adults [77].

Another challenge to both study initiation and implementation may be the ongoing (or re-emerging) restrictions due to the Corona pandemic. In addition to constraints on the conduct of research projects (e.g. [78]), the longer-term work-related perspectives and participation opportunities of people with mental illness seem to have been additionally worsened under Corona conditions [61]. Moreover, of course, many young individuals have reported being highly burdened by the pandemic [79] and experiencing more mental health problems [80].

A methodological limitation of the present study is the use of a single group pre-post design, because we will not be able to control for potential confounders. In order to have something similar to a control group, we will compare the data from participants of the IPS coaching with data of the federal average or data of standard labour market policy measures. Wherever possible, not only intra-group changes will be analysed, but also comparisons with published norms of the test procedures as well as results of other studies will be used. Nevertheless, a RCT study design should be a future approach.

In sum, the presented StAB program will contribute to the wider dissemination and implementation of Supported Employment and Education for mentally ill adolescents. More specifically, it is intended to gain improved knowledge on the application of individualized placement and support in a sample of young people with different mental health problems in Germany. Beyond improving the young people’s participation in education or working life, and consequently their career prospects, StAB should also lead to positive changes in non-work-related characteristics, such as the adolescents’ psychosocial functioning, quality of life or self-efficacy.

## Supporting information

S1 Checklist SPIRIT 2013 checklist. (PDF)

S1 Fig (TIF)

S1 Protocol (PDF)

S2 Protocol (PDF)
