# Supplementary figures and images for: Evaluation of a supported education and employment program for adolescents and young adults with mental health problems: A study protocol of the StAB project
Source: PLoS One. 2022 Jul 29;17(7):e0271803. doi: 10.1371/journal.pone.0271803 (PMC9337640; doi:10.1371/journal.pone.0271803)

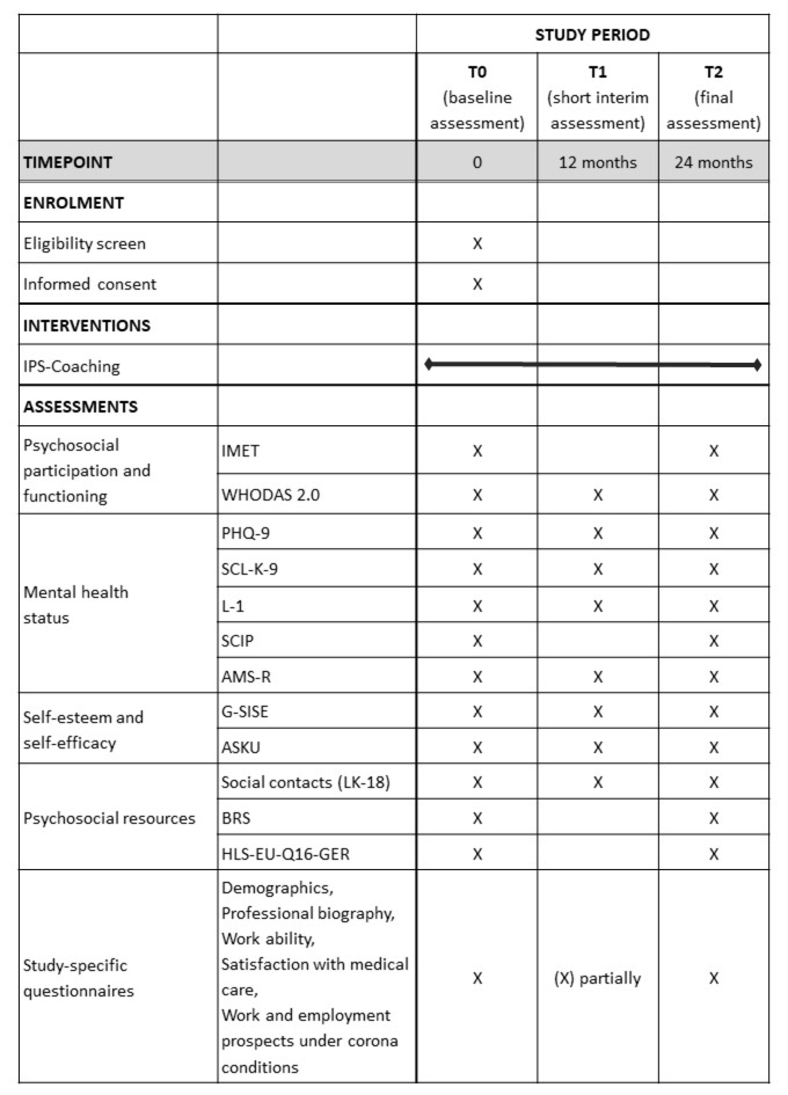

Supplement: S1 Fig — (TIF) [file pone.0271803.s002.tif]
