# Supplementary material for: Evaluation of a supported education and employment program for adolescents and young adults with mental health problems: A study protocol of the StAB project
Source: PLoS One. 2022 Jul 29;17(7):e0271803. doi: 10.1371/journal.pone.0271803 (PMC9337640; doi:10.1371/journal.pone.0271803)
Supplement: S1 Protocol — (PDF) [file pone.0271803.s003.pdf]

Application form for non-medical research projects

Procedure
Please complete the application form digitally (not handwritten), outlining the methods and objectives of the research project in as layman's terms as possible. All relevant sections of the application form should be completed. In addition, all other documents related to the study, such as promotional materials, education/consent forms, study protocol, and questionnaires, should be submitted along with the completed application form. The application form should be signed by the applicant and stamped by the institution.

File number: 2022-037-f-S

Revised version after feedback by the Ethics Committee on February 9, 2022

Status: 23/02/2022 (Version 2 / V2) - positive vote of the EC on 02.03.22 (see attachment).

<table>
<tr><th colspan="6">1. Applicant details</th></tr>
<tr><td>1.1</td><td colspan="2">Name, first name, academic degrees</td><td colspan="3">Dr. rer. nat. Lorenz Dehn, M.Sc.-Psych.,
Psychological Psychotherapist</td></tr>
<tr><td>1.2</td><td colspan="2">Address, telephone number, fax if applicable (for queries regarding the application)</td><td colspan="3">AG Psychosocial Care & Participation Research,
University Department of Psychiatry & Psychotherapy,
University Hospital OWL of the University of Bielefeld,
Evangelisches Klinikum Bethel (EvKB),
Remterweg 69/74, 33617 Bielefeld
Phone: 0521 / 772 78517</td></tr>
<tr><td>1.3</td><td colspan="2">E-mail address
By providing an email address, you consent to us sending you unencrypted emails regarding your application.</td><td colspan="3">Lorenz.Dehn@evkb.de</td></tr>
<tr><td colspan="6">1.4 Cooperation partner</td></tr>
<tr><td>1.4.1</td><td>Name, first name, academic degrees</td><td>Psaar, Gabriele</td><td>1.4.1</td><td>Name, first name, academic degrees</td><td>Evers, Christina</td></tr>
<tr><td>1.4.2</td><td>Address, phone number</td><td>v. Bodelschwingh-sche Stiftungen Bethel, foundation area proWerk Nazarethweg 4 33617 Bielefeld, Phone: +49 157 51464190</td><td>1.4.2</td><td>Address, phone number</td><td>Jobcenter Arbeitplus Bielefeld, Herforder Str. 67, 33602 Bielefeld, Phone: 0521 / 55617 33611</td></tr>
<tr><td>1.4.3</td><td>Function</td><td>Operational project coordination</td><td>1.4.3</td><td>Function</td><td>Applicant for the model project</td></tr>
</table>

2. Details of the research project

2.1 Title	**Start in education and employment (StAB)**
2.2 Project description (Please outline the background, research question, and objective of the research project)	The model project StAB (German: Start in Ausbildung und Beruf) aims at improving the integration of severely mentally ill young people up to 25 years of age into the first labour market by testing the application of Individual Placement and Support (IPS) coaching directly from the treatment setting. This innovative intervention to be tested here is based on the evidence-based Supported Education Approach (SEA), which is about school/vocational qualification for the first labour market, especially through (vocational) education measures in regular structures (e.g., school education, vocational training) and the transition to gainful employment according to the "first-place-then-train" principle (among others Hofmann/Schaub 2016; Hoffmann/Richter 2018; Maru et al. 2018; Kane et al. 2016). This approach can be considered equivalent to the Supported Employment (SE) approach for adults in terms of its structure and objective, namely direct, permanent placement in and qualification for the primary labour market, but focuses primarily on career entry. For the target group of mentally ill adolescents, a conceptual addition of therapeutic modules takes place in some cases (Hoffmann/Richter 2018). Here, SEA is consistently transferred to severely mentally ill adolescents aged 15 to 25. In accordance with § 15a SGB VI, the aim is to increase the chance of entering working life despite severe mental illness. "Individual Placement and Support" (IPS) as the best evaluated form of SE/ SEA is applied by accompanying the participants by appropriately qualified coaches - in principle not limited in time -, placing them as soon as possible in a (training) place and immediately training or qualifying them there.<br>The quasi "lifeworld coach" as a continuous, reliable reference person of the participant is located at the Bethel Foundation, proWerk, a psychosocial service provider that takes over the individual case management and support. The focus of the coaching is on the topics of coping with everyday life, work, education and career, but also on other individual needs and topics depending on the individual case and is basically oriented to the "normal" living environment of the

individual. The coach should, of course, make use of conventional rehabilitation services for individual planning.

Due to the model character of the project, the coaching phase ends 2 to max. 2.5 years after the individual start and, if possible, is transferred to a suitable follow-up perspective in individual cases if there is further need.

Another component of the project is the establishment of a competence network with all relevant actors from the fields of child and youth work, school, training, vocational participation and work as well as the psychosocial and psychiatric system. In a cooperation agreement, the criteria for cross-sectoral cooperation and long-term networking of the target group as well as strategies for the creation of structural-regional structures beyond the end of the project are laid down. The client of the project is the Jobcentre Arbeitsplus Bielefeld. The scientific accompanying research is carried out by the research department of the Clinic for Psychiatry and Psychotherapy.

The following are the primary objectives of the research project:

- Through the model intervention IPS-Coaching, at least 40% of the mentally ill adolescents participating in IPS-Coaching have started or resumed school education or vocational training or a specific preparatory internship or are gainfully employed in the primary labour market within 2 years of the start of IPS.
- As a result of the IPS coaching, the proportion of mentally ill participants who have applied for admission to a sheltered workshop (entrance procedure, vocational training area) within 2 years of the start of the IPS is lower than the corresponding rates of the national average.
- Through the model intervention IPS-Coaching, the opportunities for choice and participation in education and in working life, and thus the occupational prospects of the participating adolescents with mental illnesses, have increased within two years from the start of the IPS, so that in the before-after comparison of the intervention a diversity in employment constellations of the target group becomes clear and the increased opportunities for choice and participation are also assessed as such by the participants.

nslation

	- Within 2 years from the start of IPS coaching in conjunction with parallel (adolescent) psychotherapeutic treatment and the use of the individual budget, non-work-related characteristics have also improved in comparison to the data collection at the start of coaching, e.g. quality of life, psychopathological symptoms, neuropsychological and psychosocial functions, self-esteem and self-efficacy expectations, and subjective motivation. These included quality of life, need for inpatient treatment, psychopathological symptoms, neuropsychological and psychosocial functions, self-esteem and self-efficacy expectations, occupational motivation, and subjective expectations of participation and rehabilitation.<br>- Within two years after the start of the project, a competence network has emerged in the regions of the participating care clinics with all relevant actors in the fields of child and youth work, school, training, vocational participation and work as well as the psychosocial and psychiatric system<br>- Review / modification of the IPS coaching concept for adolescents (15-25 years), if necessary.
2.3 Description of the type of study<br>(Is it qualitative or quantitative research? How is the data collection and analysis carried out?)	The study project applied for here will be conducted as a **prospective evaluation study in a mixed-methods design**.<br>The **quantitative research focus** is on determining key figures about the intervention within the participant group and testing the intervention effects in a two-year, uncontrolled observational study in a pre-post design with two project sites. The data collection in the form of individual surveys at the beginning and end of the intervention includes, on the one hand, retrospective and cross-sectional data on the course of the disease, on the (educational) biography as well as the previous professional career, and on the other hand, above all, validated and established test procedures and examination instruments on the various psychosocial and health-related target variables of the project (see above), e.g. quality of life, psychopathological symptomatology, neuropsychological and psychosocial functions, self-esteem, professional motivation and self-efficacy expectations and subjective participation expectations. The data will be evaluated within the framework of quantitative-statistical

		analyses using established computer-assisted evaluation programs (e.g. SPSS or R). In particular, pre-post comparisons and follow-up evaluations using variance, correlation and regression analyses are planned. The analyses should include socio-demographic (e.g. age, occupational background) and clinical basic variables (e.g. ICD-10 diagnosis, psychopathological symptom severity. Depressive severity, etc.) of the participants are taken into account and, if necessary, statistically controlled.<br>The **qualitatively oriented research area** concerns, among other things, the establishment of a competence network (see above) including a corresponding evaluation with regard to actual and target analysis. For data collection, primarily qualitative (semi-)structured interviews and questionnaires with stakeholders and actors from the psychiatric and psychosocial networks are planned. Structures, working methods, effectiveness and quality of the cooperation as well as the benefit for the clients of the regional system are to be recorded. Data evaluation will be carried out using specialized computer software (e.g. MAXQDA) and will be based on the theoretical concepts of grounded theory and qualitative content analysis according to Mayring.
2.4	Is a questionnaire used?	☒ yes (Please submit a copy.)<br>→ The copy submitted in the attachment contains a first draft of the preliminary questionnaire package, which will be further developed and completed during the first project phase.<br>☐ no
2.5	Start date / duration of the research project<br>from month/year<br>until month/year (publication/final report)	01/04/2022 until 31/10/2026
2.6	Where will the project and data collection take place?	The study project will be coordinated from Bielefeld and data collection will take place at the two project sites Bielefeld (Clinic for Psychiatry and Psychotherapy/EvKB, Jobcentre Bielefeld) and Dortmund (LWL Elisabeth-Clinic, Jobcentre Dortmund). Data collection will primarily take place on the premises of the participating project partners, but if the health and living situation of the study participants does not allow it, outreach interviews in the home environment or telephone and mail surveys may also take

		place.
2.7	Who is funding the project?<br>If applicable, please name sponsors, funding organizations and attach the relevant agreements or grant notifications or contracts.<br>**Your application cannot be processed without funding information.**	☐ Funding/support commercially by:<br>(please specify here, e.g. client/sponsor)<br><br>☒ Funding/support public/non-profit by:<br>(please specify here, e.g. BMBF, DFG or similar)<br>**Federal Ministry of Labour and Social Affairs (BMAS): Federal program "Innovative ways to participate in working life - rehapro"**<br>(see forwarding notice in the appendix)<br><br>☐ Not funded, funded by the following (please specify here):
2.8	Are there any legal requirements (if necessary, consult your institution's legal department)?	N/A
**3. Details of the study participants**		
3.1	Quantity	75-80 people
	What is the rationale for the selected case number? Present the case number planning with hypotheses and evaluation methodology:	At both implementation sites, definitely 60 persons (ideally 2 x 30) are to undergo the project and the complete IPS coaching.<br>With regard to the primary quantitative target parameters (quality of life, self-efficacy, realization of participation), an improvement in the self-assessed questionnaire results between the initial and final survey is expected over the two-year (StAB) intervention period. As a statistical evaluation method, a (paired) t-test for dependent samples is basically used for this purpose, since the investigation of mean differences is only based on one (intervention) group with repeated (pre-post) questioning. With regard to the expected effect size, a small effect was initially assumed (d=0.4), since comparable empirical studies in the research field are lacking so far. Considering this effect size of d=0.4, an α-(error) level of 0.05, and a β-level of 0.2 (or a power of 0.8), the a priori case number calculation using the program G*Power 3.1 (Faul et al. 2017) resulted in a required sample size of n=52 persons. In order to

		be able to guarantee this and assuming a dropout rate of at least 20 percent in the period of 24 months after recruitment, at least 75 persons should definitely be included in the study for IPS coaching.
	Evaluation study	yes
	Case study	no
3.2	Age	15 to 25 year olds
	Minors (differentiate further if necessary)	Participants aged 15 and over who are minors and still of compulsory school age are also to be included in the study, provided that their parents have given their informed consent. Another prerequisite is the existence of or the initiation of ALG II receipt.
	18 – 40 years old	exclusively up to the age of 25.
	40 – 60 years old	-
	60 – 80 years old	-
	Older than 80 years	-
3.3	Gender	☒ male<br>☒ female
3.4	How will the study participants be recruited? (Please attach recruitment material)	The access groups can thus be categorised as follows:<br>1a - in psychiatric treatment (or in the last 12 months) and in receipt of ALG II.<br>1b - currently undergoing psychiatric treatment and in the process of receiving ALG II.<br>2a - in receipt of ALG II and in the last year of psychiatric treatment<br>2b - in receipt of ALG II and in the process of psychiatric treatment<br>The recruitment of the study participants at both project locations is therefore carried out on the one hand by the referring psychiatric clinics and on the other hand by the responsible job centres. In these facilities, all relevant employees are informed about the project comprehensively and at an early stage, e.g. in the form of departmental or ward rounds, and receive written information documents (project information, inclusion criteria, schedules), which are still being prepared in the first (preparatory) phase of the project before

		recruitment begins.
3.5	Non-consenting persons (e.g. children)	Participants over the age of 15 who are minors and still of school age are also to be included in the study, provided that informed consent has been obtained from their legal guardians. Another prerequisite is the existence of or the initiation of ALG II receipt.
3.6	What inclusion criteria have you set? (complete list)	Main eligibility criteria:<br>- Eligible participants are:<br>- Adolescents and young adults (from the age of 15 to 25 years)<br>- with a psychiatric illness, regardless of the diagnosis,<br>- who are currently receiving ALG II benefits or are entitled to such benefits.<br>- Informed written consent to participate in the evaluation study has been obtained (in the case of minors, also from their legal guardians).<br><br>Additional inclusion criteria:<br>- The participants are sufficiently competent in the spoken and written German language.<br>Migrants have an unlimited right of residence in Germany.
3.7	What exclusion criteria have you set? (complete list)	Exclusion criteria are:<br>- a currently active substance dependence<br>- an existing underachievement.
3.8	Should the trial participants be paid (expense allowance, travel expenses, etc.)? If so, what is the amount?	The participants of the job coaching do not receive any financial compensation.<br>For the qualitative sub-project, those persons who take part in in-depth individual interviews, for example, are to be provided with an expense allowance for travel, parking costs, etc., if necessary. This allowance will not exceed 10 euros per hour.

4. Risks

4.1	Are there risks for the study participant or the researcher? (if applicable, also data protection risks)	No. Psychological stress caused by questionnaires and tests is very unlikely to be a potential risk. This assessment of a low probability of occurrence of stress is based on many years of experience of researchers in dealing with questionnaires and test procedures in corresponding clinical studies. The scientific literature does not report any increased psychological distress of participants during questionnaire-based

nslation

		observational studies (e.g. Jorm et al. 2007: Participant distress in psychiatric research: a systematic review. Psychological Medicine; 37:917-926; Jaffe et al. 2015: Does it hurt to ask? A meta-analysis of participant reactions to trauma research. Clinical Psychology Review; 40:40-56; Dehn et al. 2022: Participating in longitudinal observational research on psychiatric rehabilitation: Quantitative results from a patient perspective study. Frontiers in Psychiatry).
4.2	What precautions are taken to minimise the risk?	If there are indications that the survey is stressful for the participants, they will of course first be offered a break or the survey will be interrupted and, if necessary, resumed in a modified form. The participants will be explicitly informed in the information letter as well as at the beginning of the survey(s) about the possibilities to take breaks, to skip parts of the survey or to stop the survey without disadvantages.

5. Ethical aspects / due process

5.1	Submitting Informed Consent	see attachments → the attached information, data protection and consent forms were prepared on the basis of templates provided by the Ethics Committee of Bielefeld University.
5.2	Dealing with non-consenting persons (e.g. children)	See above (3.5)
5.3	How do you ensure that the applicable data protection regulations (BDSG, DSG NRW, GDGS NRW) are complied with?	The processing of personal data is always subject to the data protection regulations of the Data Protection Ordinance, the BDSGV and the laws of the state of North Rhine-Westphalia in the currently valid version. In addition, all academic project staff are bound by the Data Protection Act of the Protestant Church in Germany (DSG-EKD). The handling of the collected data is explained in detail in the clarification and data protection information (see appendix).<br>The questionnaires to be completed and the data collected will be pseudonymised, i.e. encrypted with a code number, so that neither the name nor the date of birth of the participant will become known during data processing. The necessary assignment list is stored for the duration of the study in the form of a digital coding list, which is protected with a password known only to the project leader. After completion of the study survey, all data will be digitised in anonymised form and digitally archived in the internal data backup system of the Evangelisches Krankenhaus

Translation

		Bielefeld. Any paper data accumulated until then will be destroyed by a special service provider in compliance with data protection regulations.
5.4	Are auditory or photographic recordings of the study participants foreseen? If so, how will these recordings be handled during and after the study?	For the qualitative sub-project, it is planned to make an audio recording of the individual interviews using a portable audio recorder, which will then be written down (i.e. "typed" as text) for scientific evaluation and then deleted. Until then, the audio recording is stored digitally on the audio recorder, which is kept in the safe of the archive of the research department of the Department of Psychiatry and Psychotherapy. The transcribed interview will be stored as a digital text document and exclusively in anonymised form in the digital data backup system of the Evang. Klinikum Bethel. In accordance with the documents presented in the appendix, a corresponding declaration of clarification, data protection and consent will of course also be drawn up for the qualitative sub-project.
5.5	Has a volunteer insurance policy been taken out for the benefit of the trial participants? (If yes, please enclose confirmation of insurance and general conditions of insurance**)**	No.<br>The research project to be reviewed here is a non-medical, health care research-related observational study that is not associated with increased health risks. The participants are also explicitly informed that their participation is voluntary (see appendices).

6. Final declarations

**6.1 Repeat the title of the project here**	
**Start in education and employment (StAB)**	
**6.2 Signature of the applicant(s), stamp of the institution**<br>(in the case of several responsible project participants, signatures of all participants)	
**name, date, signature, stamp**	**name, date, signature, stamp**
**name, date, signature, stamp**	**name, date, signature, stamp**

6.3. Declaration of consent of the director of the clinic, department of the institute
I have been informed by the project leader about the study planned in my institution and agree to its implementation. (Signature, name in block capitals and stamp)

name, date, signature, stamp

6.4. If applicable, declaration of power of attorney if the application is (also) submitted for third parties (e.g. by study centres, sponsors, contract research organisations/CROs, etc.):

I affirm that I have been commissioned and authorised to submit to the Ethics Committee on behalf of the participants / physicians to be advised. I am aware that the Ethics Committee may request the submission of powers of attorney.
I undertake to bring the results of any consultation to the attention of all parties involved.

name, date, signature

Translation

7. Explanation of fees:

The Medical Association of Westphalia-Lippe charges fees for the activities of the Ethics Committee in accordance with the Administrative Fees Regulations in the currently valid version. The party liable for the fees is the applicant, i.e. in the case of a consultation in accordance with § 15 Para. 1 of the Professional Code of Conduct of the Medical Association of Westphalia-Lippe, as a rule the doctor responsible for the research project.

If nothing to the contrary is stated here, the fee notice shall be issued to the responsible project manager named in section 1.1.

Please ensure that field 2.7 on funding is completed.

**Different invoice recipient:**	☒ YES ☐ NO
The fee notice is to be issued to:<br><br>Company<br>Department<br>Contact<br>Address<br><br>Additional information, if applicable	**Evang. Klinikum Bethel (EvKB)<br>University Department of Psychiatry & Psychotherapy,<br>University Hospital OWL of the University of Bielefeld,<br>Remterweg 69/74, 33617 Bielefeld<br>Prof. Dr. Martin Driessen (chief physician)<br>Remterweg 69-71, 33617 Bielefeld**
The fee notice addressed in this way should be sent to:<br><br>Company<br>Department<br>Contact<br>Address<br><br>Additional information, if applicable	**Evang. Klinikum Bethel (EvKB)<br>University Department of Psychiatry & Psychotherapy,<br>University Hospital OWL of the University of Bielefeld,<br>Remterweg 69/74, 33617 Bielefeld<br>Prof. Dr. Martin Driessen (chief physician)<br>Remterweg 69-71, 33617 Bielefeld**

Translation

Patient Protection | Freedom of research

ETHICS COMMITTEE
of the Medical Association of Westphalia-Lippe and
the Westphalian Wilhelms-University of Münster

Ethik-Kommission Münster - Gartenstraße 210-214 – 48147 Münster

Mr.
Dr. rer. nat. Lorenz Dehn, M.Sc.-Psych.
Evangelisches Klinikum Bethel (EvKB)
Universitätsklinikum OWL der Universität
Bielefeld, Universitätsklinik für Psychiatrie &
Psychotherapie
Remterweg 69/74
33617 Bielefeld

Email only:

Lorenz.Dehn@evkb.de

March 2, 2022 CS

**File number (please always indicate):**	**2022-037-f-S**
Title of the research project:	Start in education and employment (StAB)
Here:	01_new application as responsible EC, receipt 17/01/2022

Dear Dr. Dehn,

for the above-mentioned research project, you have requested the advice of the Ethics Committee. The Ethics Committee has deliberated on your application, also considering supplementary/revised documents and decided:

The Ethics Committee has no fundamental ethical or legal objections against the implementation of the research project.

The present assessment applies to the research project as it is presented on the basis of the documents mentioned in Annex 1.

For the decision of the Ethics Committee, the Medical Association of Westphalia-Lippe charges fees in accordance with its schedule of administrative fees. A separate notice will be issued regarding the fees.

General information:

With the present statement, the members of the Ethics Committee advises the physicians who are members of the medical association of Westphalia-Lippe on the questions of professional ethics and professional law associated with the research project in accordance with § 15 Paragraph 1 of the Professional Code of Conduct of the Medical Association of Westphalia-Lippe (ÄKWL).

Translation

If you have submitted the application on behalf of other physicians involved, you are obligated to inform all participating physicians of the results of this consultation.

The assessment of the Ethics Committee, as an open-ended consultation, is not binding for the applicant. Irrespective of the present opinion, the medical, ethical and legal responsibility for the implementation of the research project remains with its director, client and all physicians involved in the project.

The members of the Ethics Committee listed in Annex 2 took part in the deliberations and resolutions.
The Ethics Committee of the Medical Association of Westphalia-Lippe and the Westphalian Wilhelms-University of Münster is organized and works according to the legal regulations and the GCP guidelines of the ICH.

Yours sincerely

Prof. Dr. phil. Christiane Frantz
Deputy Chairwoman of the Ethics Committee

Translation

Appendix 1

The following documents have been submitted to the Ethics Committee:

In the case of multiple versions of a document, the evaluation refers to the most recent:

Receipt	Date	Attachment
17.01.2022	17.01.2022	Initial application Start in education and occupation (StAB)
17.01.2022	17.01.2022	StAB_1_EK_Application letter
17.01.2022	17.01.2022	StAB_2_EK_Application_Complete
17.01.2022	17.01.2022	StAB_3_EK_Anlage1_Aufkl_Einwill_Datensch_Teilnehmende
17.01.2022	17.01.2022	StAB_3_EK_Anlage2_Aufkl_Einwill_Datensch_Sorgeberecht
17.01.2022	17.01.2022	StAB_3_EK_Anlage4_Zuwendungsbescheid_Jobcenter-EvKB
17.01.2022	17.01.2022	StAB_3_EK_Annex3_Questionnaires_Draft
17.01.2022	17.01.2022	StAB_4_EK_CV_Project_manager_Dehn_2022
02/24/2022	02/24/2022	AW Ethics Application Start in Education and Employment (StAB) our file number 2022-037-f-S
24.02.2022	24.02.2022	StAB_EKMS_Formular_nicht-ärztliche-Forschungsvorhaben_V2-22-02-23

Translation

Appendix 2

The following members of the Ethics Committee took part in the deliberations and decision-making process:

Physicians

Prof. Dr. med. Dirk Föll, Münster

Univ. Prof. Dr. med. Karin Hengst, Münster

Prof. Dr. med. Heinrich Schulze Mönking, Telgte

PD Dr. med. Dirk Wähnert, Bielefeld

Dentists

PD Dr. med. dent. Anne Wolowski, Münster

Pharmacists

Hans-Theo Fortmeier, Havixbeck

Persons with experience in the field of experimental design and statistics

Dr. rer. nat. Joachim Gerß Dipl. -Stat., Münster

Persons qualified to hold judicial office / lawyers

Dr. Sascha Rolf Lüder, Düsseldorf, Germany

Persons with scientific experience in the field of ethics in medicine

Reverend Frank Neumann, Münster

Persons from the field of patient advocacy; laypersons

Annette Hünefeld Dipl. ped., Bonn

Other disciplines

Michael Finke Dipl.-Päd., MAE, Osnabrück

Prof. Dr. phil. Christiane Frantz, Münster

Translation
