# Supplementary material for: Evaluation of a supported education and employment program for adolescents and young adults with mental health problems: A study protocol of the StAB project
Source: PLoS One. 2022 Jul 29;17(7):e0271803. doi: 10.1371/journal.pone.0271803 (PMC9337640; doi:10.1371/journal.pone.0271803)
Supplement: S2 Protocol — (PDF) [file pone.0271803.s004.pdf]

Antragsformular für nicht-ärztliche Forschungsvorhaben

Vorgehensweise
Bitte füllen Sie das Antragsformular digital (nicht handschriftlich) aus und stellen Sie die Methoden und Ziele des Forschungsprojektes in möglichst laienverständlicher Form dar. Alle relevanten Abschnitte des Antragsformulars sollten ausgefüllt werden. Außerdem sind alle weiteren mit der Studie in Zusammenhang stehenden Dokumente, wie z.B. Werbematerial, Aufklärung / Einwilligungserklärung, Studienprotokoll und Fragebögen, gemeinsam mit dem ausgefüllten Antragsformular einzureichen. Das Antragsformular sollte von dem Antragsteller unterschrieben und mit einem Stempel der Einrichtung versehen werden.

Aktenzeichen: 2022-037-f-S

Überarbeitete Version nach Rückmeldung durch die Ethik-Kommission vom 9. Februar 2022

Stand: 23.02.2022 (Version 2 / V2) → positives Votum der EK am 02.03.22 (siehe Anhang)

1. Angaben zum Antragsteller

1.1	Name, Vorname, akademische Grade	Dr. rer. nat. **Lorenz Dehn,** M.Sc.-Psych.,<br>Psychologischer Psychotherapeut
1.2	Adresse, Telefonnummer, ggf. Fax<br>(für Rückfragen bezüglich des Antrags)	AG Psychosoziale Versorgungs- & Teilhabeforschung,<br>Universitätsklinik für Psychiatrie & Psychotherapie,<br>Universitätsklinikum OWL der Universität Bielefeld,<br>Evangelisches Klinikum Bethel (EvKB),<br>Remterweg 69/74, 33617 Bielefeld<br>Tel.: 0521 / 772 78517
1.3	E-Mail-Adresse<br>Mit der Angabe einer E-Mail-Adresse erklären Sie sich damit einverstanden, dass wir Ihnen unverschlüsselte E-Mails zu Ihrem Antrag senden.	Lorenz.Dehn@evkb.de

1.4 Kooperationspartner

1.4.1	Name, Vorname, akademische Grade	Psaar, Gabriele	1.4.1	Name, Vorname, akademische Grade	Evers, Christina
1.4.2	Adresse, Telefonnummer	v. Bodelschwingh-sche Stiftungen Bethel, Stiftungs-bereich proWerk Nazarethweg 4 33617 Bielefeld, Tel: +49 157 51464190	1.4.2	Adresse, Telefonnummer	Jobcenter Arbeit*plus* Bielefeld, Herforder Str. 67, 33602 Bielefeld, Tel: 0521 / 55617 33611
1.4.3	Funktion	Operative Projekt-koordination	1.4.3	Funktion	Antragstellerin für das Modell-projekt

2. Angaben zum Forschungsprojekt	
2.1 Titel	**Start in Ausbildung und Beruf (StAB)**
2.2 Projektbeschreibung (Bitte skizzieren Sie Hintergrund, Fragestellung und Ziel des Forschungsvorhabens)	Das Modellprojekt StAB (Start in Ausbildung und Beruf) zielt auf eine Verbesserung der Integrationsarbeit auf den 1. Arbeitsmarkt mit schwer psychisch kranken jungen Menschen bis 25 J. ab, indem direkt aus dem Behandlungssetting heraus zeitgleich die Anwendung des *Individual Placement and Support (IPS)* – Coachings erprobt wird. Diese hier zu erprobende, innovative Intervention basiert auf dem evidenzbasierten *Supported Education Approach (SEA*), bei dem es um die schulische/berufliche Qualifizierung für den ersten Arbeitsmarkt, insbesondere durch (Berufs-)Bildungsmaßnahmen in Regelstrukturen (z.B. Schulausbildung, Berufsausbildung) und den Übergang in die Erwerbsarbeit nach dem *„First-place-then-train*“-Prinzip geht (u.a. Hofmann/Schaub 2016; Hoffmann/Richter 2018; Maru et al. 2018; Kane et al. 2016). Dieser Ansatz ist von seinem Aufbau und der Zielstellung, nämlich der direkten, dauerhaften Platzierung auf dem und Qualifizierung für den ersten Arbeitsmarkt, äquivalent zum Ansatz des *Supported Employment* (SE) für Erwachsene zu betrachten, fokussiert jedoch primär auf den Berufseinstieg. Für die Zielgruppe psychisch kranker Adoleszenter findet dabei teilweise eine konzeptionelle Ergänzung durch therapeutische Module statt (Hoffmann/Richter 2018). SEA wird hier konsequent übertragen auf schwer psychisch kranke Adoleszent*innen von 15 bis 25 Jahren. Entsprechend § 15a SGB VI soll die Chance auf den Eintritt in das Erwerbsleben trotz schwerer psychischer Erkrankung erhöht werden. „Individual Placement and Support“ (IPS) als die am besten evaluierte Form des SE/ SEA, wird angewandt, indem die Teilnehmenden durch entsprechend qualifizierte Coaches - zeitlich im Grundsatz nicht limitiert - begleitet, möglichst zeitnah auf einem (Aus-)Bildungsplatz platziert und sogleich dort trainiert bzw. qualifiziert werden.<br>Der quasi „Lebenswelt-Coach“ als kontinuierliche, zuverlässige Bezugsperson des Teilnehmenden ist bei der Stiftung Bethel, proWerk verortet, einem psychosozialen Leistungsanbieter, der die Einzelfallsteuerung und -begleitung übernimmt. Der Fokus des

	Coachings liegt bei den Themen Alltagsbewältigung, Arbeit, Bildung und Beruf, aber auch bei weiteren individuellen je nach Einzelfall bestehenden Bedarfen und Themen und ist grundsätzlich an der „normalen" Lebenswelt der*des Einzelnen orientiert. Der Coach soll selbstverständlich die konventionellen Reha-Angebote für die individuelle Planung mitnutzen.<br>Aufgrund des Modellcharakters endet im Projekt die Coaching–Phase 2 bis max. 2,5 Jahre nach individuellem Beginn und wird im Einzelfall bei weiterem Bedarf möglichst in eine geeignete Anschlussperspektive übergeleitet.<br>Weiterer Bestandteil des Projektes ist der Aufbau eines Kompetenznetzwerks mit allen relevanten Akteuren aus den Bereichen Kinder- und Jugendarbeit, Schule, Ausbildung, berufliche Teilhabe und Arbeit sowie dem psychosozialen und psychiatrischen System. In einer Kooperationsvereinbarung werden die Kriterien für die sektorenübergreifende Zusammenarbeit und langfristige Vernetzung der Zielgruppe sowie Strategien für die Schaffung strukturell-regionaler Strukturen über das Projektende hinaus festgeschrieben. *Auftraggeber* des Projektes ist das Jobcenter Arbeitsplus Bielefeld. Die *wissenschaftliche Begleitforschung* erfolgt durch die Forschungsabteilung der Klinik für Psychiatrie und Psychotherapie.<br>Folgende **Ziele** liegen dem Forschungsprojekt primär zugrunde:<br>- Durch die Modell-Intervention IPS-Coaching haben mindestens 40% der am IPS- Coaching teilnehmenden psychisch kranken Adoleszent*innen, innerhalb von 2 Jahren ab Beginn des IPS, eine Schulbildung bzw. berufsqualifizierende Ausbildung oder ein spezifisch vorbereitendes Praktikum begonnen bzw. wiederaufgenommen oder gehen einer Erwerbstätigkeit auf dem ersten Arbeitsmarkt nach.<br>- Durch das IPS-Coaching ist der Anteil psychisch kranker Teilnehmer*innen, die innerhalb von 2 Jahren ab Beginn des IPS einen Antrag auf die Aufnahme in eine WfbM (Eingangsverfahren, Berufsbildungsbereich) gestellt haben, geringer als die entsprechenden Quoten des Bundesdurchschnitts ausweisen.

	- Durch die Modell-Intervention IPS-Coaching haben sich die Wahl- und Teilhabemöglichkeiten an Bildung bzw. am Arbeitsleben und damit einhergehend die beruflichen Perspektiven der teilnehmenden Adoleszent*innen mit psychischen Erkrankungen, innerhalb von 2 Jahren ab Beginn des IPS, vergrößert, sodass sich im Vorher-Nachher-Vergleich der Intervention eine Diversität in Beschäftigungs-konstellationen der Zielgruppe verdeutlicht und die gestiegenen Wahl- und Teilhabemöglichkeiten durch die Teilnehmer*innen auch als solche eingeschätzt werden.<br>- Durch das IPS-Coaching in Verbindung mit der parallelen (jugend-)psychotherapeutischen Behandlung und dem Einsatz des individuellen Budgets haben sich innerhalb von 2 Jahren ab Beginn des IPS über die berufliche Teilhabe (Erreichter (Aus-)Bildungsabschluss/ beruflicher Status und Zufriedenheit der Adoleszent*innen mit diesem) hinaus auch nicht-arbeitsbezogene Merkmale, im Vergleich zur Datenerhebung zu Beginn des Coachings, verbessert, z.B. Lebensqualität, teilstationäre/ stationäre Behandlungsnotwendigkeit, psychopathologische Symptomatik, neuropsychologische und psychosoziale Funktionen, Selbstwertgefühl und Selbstwirksamkeitserwartung, berufliche Motivation und subjektive Teilhabe-/Rehabilitationserwartung.<br>- Innerhalb von zwei Jahren nach Beginn des Projektes ist ein Kompetenznetzwerk in den Regionen der beteiligten Versorgungskliniken mit allen relevanten Akteuren in den Bereichen Kinder- und Jugendarbeit, Schule, Ausbildung, berufliche Teilhabe und Arbeit sowie dem psychosozialen und psychiatrischen System entstanden<br>- Überprüfung / ggf. Modifizierung des IPS-Coaching-Konzepts für Adoleszent*innen (15-25 Jahre)
2.3 Beschreibung des Studientyps (Handelt es sich um qualitative oder quantitative Forschung? Wie erfolgt die	Das hier beantragte Studienprojekt wird als **prospektive Evaluationsstudie im Mixed-Methods-Design** durchgeführt.<br>Der **quantitative Forschungsschwerpunkt** fokussiert dabei auf die Ermittlung von Kennzahlen über die Intervention innerhalb der Teilnehmendengruppe und die Überprüfung der Interventionseffekte in einer zweijährigen, unkontrollierten Beobachtungsstudie im Prä-

	Datenerhebung und Auswertung?)	Post-Design mit zwei Projektstandorten. Die Datenerhebung in Form von Einzelbefragungen zu Beginn und Ende der Intervention umfasst einerseits retrospektive und Querschnittsdaten zum Krankheitsverlauf, zur (Aus-)Bildungsbiografie sowie der bisherigen Berufslaufbahn, andererseits vor allem validierte und etablierte Testverfahren und Untersuchungsinstrumente zu den unterschiedlichen psychosozialen und gesundheitlichen Zielgrößen des Projektes (s. oben), z.B. Lebensqualität, psychopathologische Symptomatik, neuropsychologische und psychosoziale Funktionen, Selbstwertgefühl, berufliche Motivation und Selbstwirksamkeits-erwartung und subjektive Teilhabeerwartung. Die Auswertung der Daten erfolgt im Rahmen quantitativ-statistischer Analysen mittels etablierter computergestützter Auswertungsprogramme (z.B. SPSS oder R), geplant sind insbesondere Prä-Post-Vergleiche sowie Verlaufsauswertungen mittels Varianz-, Korrelations- und Regressionsanalysen. Bei den Auswertungen sollen soziodemografische (u.a. Alter, Berufshintergrund) sowie klinische Basisvariablen (u.a. ICD-10-Diagnose, psychopathologische Symptomschwere. Depressivitätsgrad etc.) der Teilnehmenden Berücksichtigung finden und ggf. statistisch kontrolliert werden.<br>Der **qualitativ ausgerichtete Forschungsbereich** betrifft u.a. den Aufbau eines Kompetenznetzwerks (s.o.) einschließlich einer entsprechenden Evaluation hinsichtlich Ist- und Zielanalyse. Für die Datenerhebung sind vorrangig qualitative (semi-)strukturierte Interviews und Fragebögen mit Stakeholdern und Akteuren aus den psychiatrischen und psychosozialen Netzwerken vorgesehen. Erfasst werden sollen Strukturen, Arbeitsweise, Wirksamkeit und Qualität der Zusammenarbeit sowie der Nutzen für die Klientinnen und Klienten des regionalen Systems. Die Datenauswertung erfolgt unter Verwendung von spezialisierter Computersoftware (z.B. MAXQDA) und wird anhand der theoretischen Konzeptionen der Grounded Theory bzw. der qualitativen Inhaltsanalyse nach Mayring durchgeführt.
2.4	Wird ein Fragebogen eingesetzt?	☒ ja (Bitte reichen Sie eine Kopie ein)<br>→ Die in der Anlage eingereichte Kopie beinhaltet einen ersten Entwurf des vorläufigen Fragebogenpakets, welches in der

		ersten Projektphase weiterentwickelt und vervollständigt wird.<br>☐ nein
2.5	Startzeitpunkt / Dauer des Forschungsprojektes<br>von MM/JJJJ<br>bis MM/JJJJ (Publikation/Abschlussbericht)	01.04.2022 bis 31.10.2026
2.6	Wo wird das Projekt und die Datenerhebung stattfinden?	Das Studienvorhaben wird von Bielefeld aus koordiniert und die Datenerhebung findet an den beiden Projektstandorten Bielefeld (Klinik für Psychiatrie und Psychotherapie/EvKB, Jobcenter Bielefeld) und Dortmund (LWL Elisabeth-Klinik, Jobcenter Dortmund) statt. Die Datenerhebung wird primär in Räumlichkeiten der teilnehmenden Projektpartner stattfinden, sollte es die Gesundheits- und Lebenssituation der Studienteilnehmer*innen jedoch nicht zulassen, können ggf. auch aufsuchende Befragungstermine im häuslichen Umfeld beziehungsweise Telefon- und Postbefragungen stattfinden.
2.7	Durch wen wird das Projekt finanziert? Bitte nennen Sie ggf. Sponsoren, Förderorganisationen und legen Sie die entsprechenden Vereinbarungen oder Bewilligungsbescheide bzw. Verträge bei. **Ohne Angaben zur Finanzierung kann Ihr Antrag nicht bearbeitet werden.**	☐ Finanzierung/Förderung kommerziell durch:<br>(bitte hier angeben, z.B. Auftraggeber/Sponsor)<br><br>☒ Finanzierung/Förderung öffentlich/gemeinnützig durch:<br>(bitte hier angeben, z.B. BMBF, DFG o. ä.)<br>**Bundesministerium für Arbeit und Soziales (BMAS): Bundesprogramm "Innovative Wege zur Teilhabe am Arbeitsleben - rehapro"** (→ s. Weiterleitungsbescheid im Anhang)<br><br>☐ nicht gefördert, finanziert aus folgenden Mitteln (bitte hier angeben):
2.8	Bestehen gesetzliche Auflagen (ggf. Beratung durch Justiziariat Ihrer Einrichtung)?	N/A

3. Angaben zu den Studienteilnehmern

3.1	Anzahl	75-80 Personen

	Wie wird die gewählte Fallzahl begründet? Stellen Sie die Fallzahlplanung mit Hypothesen und Auswertungsmethodik dar:	An beiden Durchführungsorten sollen definitiv 60 Personen (idealerweise 2 x 30) das Projekt und das komplette IPS-Coaching durchlaufen.<br>In Bezug auf die primären quantitativen Zielparameter (Lebensqualität, Selbstwirksamkeit, Teilhabeverwirklichung) wird über den zweijährigen (StAB-)Interventionsverlauf hinweg mit einer Verbesserung der selbsteingeschätzten Fragebogenergebnisse zwischen der Eingangs- und Abschlussbefragung gerechnet. Als statistische Auswertungs-methodik kommt hierfür grundsätzlich ein (gepaarter) t-Test für abhängige Stichproben zum Einsatz, da sich die Untersuchung der Mittelwertsdifferenzen lediglich auf eine (Interventions-)Gruppe mit wiederholter (Prä-Post-)Befragung stützt. Hinsichtlich der zu erwartenden Effektstärke wurde zunächst von einem kleinen Effekt ausgegangen (d=0,4), da bislang vergleichbare empirische Studien in dem Forschungsfeld fehlen. Unter Berücksichtigung dieser Effektstärke von d=0,4, eines α-(Fehler)Niveaus von 0,05 sowie eines β-Niveaus von 0,2 (bzw. einer Power von 0,8) ergab die a-priori Fallzahlberechnung mittels des Programms G*Power 3.1 (Faul et al. 2017) eine benötigte Stichprobengröße von n=52 Personen. Um dies abschließend auch gewährleisten zu können und ausgehend von einer Dropout-Quote von mind. 20 Prozent in dem Zeitraum von 24 Monaten nach der Rekrutierung, sollen folglich definitiv mindestens 75 Personen für das IPS-Coaching in die Studie einbezogen werden.
	Evaluationsstudie	Ja
	Fallstudie	Nein
3.2	Alter	15 bis 25jährige Personen
	Minderjährige (ggf. weiter differenzieren)	Es sollen auch minderjährige, noch schulpflichtige Teilnehmer*innen ab 15 Jahren in die Studie eingeschlossen werden, insofern ein informiertes Einverständnis der Eltern vorliegt. Voraussetzung ist außerdem das Vorliegen bzw. die Anbahnung eines ALG-II-Bezugs.
	18 – 40 Jahre	ausschließlich bis 25 Jahre.
	40 – 60 Jahre	-
	60 – 80 Jahre	-
	älter als 80 Jahre	-

3.3	Geschlecht	☒ männlich<br>☒ weiblich
3.4	Wie erfolgt die Rekrutierung der Studienteilnehmer? (Rekrutierungs-material bitte beifügen)	Die Zugangsgruppen lassen sich so folgendermaßen kategorisieren:<br>1a - in psychiatrischer Behandlung (oder in den letzten 12 Monaten) und im ALG II-Bezug<br>1b - in aktueller psychiatrischer Behandlung, darin Anbahnung von ALG II-Bezug<br>2a - in ALG II-Bezug und im letzten Jahr psychiatrische Behandlung<br>2b - in ALG II-Bezug und in Anbahnung einer psychiatrischen Behandlung<br>Die Rekrutierung der Studienteilnehmer*innen erfolgt also an beiden Projektstandorten einerseits durch die zuweisenden psychiatrischen Kliniken als andererseits auch durch die zuständigen Jobcenter. In diesen Einrichtungen werden alle betreffenden Mitarbeitenden frühzeitig und umfassend über das Projekt informiert, z.B. in Form von Abteilungs- oder Stationsrunden, und erhalten schriftliche Informationsunterlagen (Projektinformationen, Einschlusskriterien, Ablaufpläne), die in der ersten (Vorbereitungs-)Phase des Projektes vor dem Rekrutierungsstart *noch erstellt* werden.
3.5	Nicht-Einwilligungsfähige (z.B. Kinder)	Es sollen auch minderjährige, noch schulpflichtige Teilnehmer*innen ab 15 Jahren in die Studie eingeschlossen werden, insofern das informierte Einverständnis der Sorgeberechtigten eingeholt wurde. Voraussetzung ist außerdem das Vorliegen bzw. die Anbahnung eines ALG-II-Bezugs.
3.6	Welche Einschlusskriterien haben Sie festgelegt? (vollständige Auflistung)	Haupteinschlusskriterien:<br>Teilnahmeberechtigt sind:<br>- Jugendliche und junge Erwachsene (ab dem **15. bis zum 25.** Lebensjahr)<br>- mit einer **psychiatrischen Erkrankung**, unabhängig welche Diagnose vorliegt,<br>- die sich aktuell im **Leistungsbezug ALG II** befinden bzw. Anspruch auf diesen haben.<br>- Das informierte schriftliche Einverständnis zur Teilnahme an der

		Evaluationsstudie liegt vor (bei Minderjährigen auch der Sorgeberechtigten).<br>Ergänzende Einschlusskriterien:<br>- Die Teilnehmenden sind der deutschen Sprache in Wort und Schrift ausreichend kompetent<br>- Bei Migrant*innen besteht ein unbefristetes Aufenthaltsrecht in Deutschland.
3.7	Welche Ausschlusskriterien haben Sie festgelegt? (vollständige Auflistung)	Ausschlusskriterien sind:<br>- eine aktuell aktive Substanzabhängigkeit<br>- eine vorliegende Minderbegabung.
3.8	Soll den <u>Versuchsteilnehmern</u> ein Entgelt (Aufwands-entschädigung, Fahrtkosten o.ä.) gezahlt werden? Wenn ja, in welcher Höhe?	Die Teilnehmenden des Jobcoachings erhalten keine finanzielle Aufwandsentschädigung.<br>Für das qualitative Teilprojekt sollen denjenigen Personen, die beispielsweise an vertiefenden Einzelinterviews teilnehmen, ggf. eine Aufwandsentschädigung für Anreise, Parkkosten etc. bereitgestellt werden. Diese Aufwandsentschädigung wird eine Summe von 10Euro pro Zeitstunde nicht überschreiten.
**4. Risiken**		
4.1	Bestehen Risiken für den Studienteilnehmer bzw. den Forscher? (ggf. auch Datenschutzrisiken)	Nein. Psychische Belastungen durch Fragebögen und Testverfahren sind als mögliche Risken insgesamt sehr unwahrscheinlich. Diese Einschätzung einer geringen Auftretenswahrscheinlichkeit der Belastungen basiert auf langjähriger Erfahrung der Forschenden im Umgang mit Fragebögen und Testverfahren in entsprechenden klinischen Studien. Auch in der wissenschafltichen Literatur werden insgesamt keine erhöhten psychischen Belastungen von Probanden während fragebogengestützter Beobachtungsstudien berichtet (z.B. Jorm et al. 2007: Participant distress in psychiatric research: a systematic review. *Psychological Medicine*; 37:917–926; Jaffe et al. 2015: Does it hurt to ask? A meta-analysis of participant reactions to trauma research. *Clinical Psychology Review*; 40:40–56; Dehn et al. 2022: Participating in longitudinal observational research on psychiatric rehabilitation: Quantitative results from a patient perspective study. *Frontiers in Psychiatry*)
4.2	Welche Vorsichtsmaß-	Ergeben sich Hinweise auf eine Belastung der Teilnehmer*innen durch die Befragung, wird selbstverständlich zunächst eine Pause angeboten

	nahmen werden zur Minimierung des Risikos getroffen?	bzw. die Befragung abgebrochen und ggf. modifiziert wieder aufgenommen. Die Teilnehmenden werden sowohl im Aufklärungsschreiben als auch zu Beginn der Befragung(en) explizit auf die Möglichkeiten hingewiesen, Pausen zu machen, Teile der Befragung zu überspringen oder die Befragung ohne Nachteile abzubrechen.
**5. Ethische Aspekte /ordnungsgemäße Vorgehensweise**		
5.1	Aufklärung und Einwilligung einreichen	siehe Anlagen. → Die beigefügten Aufklärungs-, Datenschutz und Einwilligungsformulare wurden auf der Grundlage von Vorlagen der *Ethikkommission der Universität Bielefeld* erstellt.
5.2	Umgang mit Nicht-Einwilligungsfähigen (z.B. Kinder)	siehe oben (3.5)
5.3	Wie stellen Sie sicher, dass die geltenden Datenschutz-bestimmungen (BDSG, DSG NRW, GDGS NRW) eingehalten werden?	Die Verarbeitungen der persönlichen Daten unterliegen grundsätzlich den datenschutzgesetzlichen Bestimmungen der Datenschutzgrundverordnung, dem BDSGV sowie den Gesetzen des Landes NRW in der aktuell gültigen Fassung. Darüber hinaus sind alle wissenschaftlichen Projektmitarbeitenden dem Datenschutzgesetz der Evangelischen Kirche Deutschland (DSG-EKD) verpflichtet. Der Umgang mit den erhobenen Daten wird ausführlich in den Aufklärungs- und Datenschutzinformationen erläutert (siehe Anhang).<br>Die auszufüllenden Fragebögen und erhobenen Daten werden pseudonymisiert, d. h. mit einer Codenummer verschlüsselt, sodass bei der Datenverarbeitung weder der Name noch das Geburtsdatum des Teilnehmenden bekannt wird. Die notwendige Zuordnungsliste wird für die Zeitdauer der Studie in Form einer digitalen Kodierliste gespeichert, die mit einem Passwort geschützt ist, welches ausschließlich dem Projektleiter bekannt ist. Nach Abschluss der Studienerhebung werden alle Daten in anonymisierter Form digitalisiert und im internen Datensicherungssystem des Evangelischen Krankenhauses Bielefeld digital archiviert. Bis dahin angefallene Daten in Papierform werden durch einen speziellen Dienstleister datenschutzkonform vernichtet.
5.4	Sind auditive oder fotografische Aufnahmen von den Studienteil-	Für das qualitative Teilprojekt ist geplant, von den Einzelinterviews eine Tonaufnahme mittels tragbaren Audiorekorder anzufertigen, die anschließend zur wissenschaftlichen Auswertung verschriftlicht (d.h. als Text „abgetippt“) und daraufhin gelöscht wird. Die Tonaufnahme wird bis

	nehmern vorgesehen? Falls ja, wie wird mit diesen Aufnahmen während der Studie und nach Studienabschluss umgegangen?	dahin digital auf dem Audiorekorder gespeichert, der im Tresorschrank des Archivs der Forschungsabteilung der Klinik für Psychiatrie und Psychotherapie aufbewahrt wird. Das verschriftlichte Interview wird als digitales Textdokument und ausschließlich in anonymisierter Form im digitalen Datensicherungssystem des Evang. Klinikums Bethel gespeichert. In Anlehnung an die in der Anlage dargestellten Dokumente wird für das qualitative Teilprojekt selbstverständlich ebenfalls noch eine entsprechende Aufklärungs-, Datenschutz- und Einwilligungserklärung erstellt.
5.5	Ist zugunsten der Versuchsteilnehmer eine Probandenversicherung abgeschlossen worden? (Wenn ja, bitte Versicherungsbestätigung und allgemeine Versicherungsbedingungen beifügen)	Nein.<br>Bei dem hier zu begutachtenden Forschungsprojekt handelt es sich um eine nicht-ärztliche, versorgungsforschungs-bezogene Beobachtungsstudie, die nicht mit erhöhten gesundheitlichen Risiken einhergeht. Die Teilnehmenden werden zudem explizit auf die Freiwilligkeit ihrer Teilnahme hingewiesen (siehe Anlagen).

6. Abschließende Erklärungen

**6.1 Titel des Vorhabens hier wiederholen**
Start in Ausbildung und Beruf – StAB

**6.2 Unterschrift des/ der Antragsteller(in/nen), Stempel der Einrichtung**<br>(bei mehreren verantwortlichen Projektbeteiligten Unterschriften aller Beteiligten)	
**Name, Datum, Unterschrift , Stempel**	**Name, Datum, Unterschrift , Stempel**
**Name, Datum, Unterschrift , Stempel**	**Name, Datum, Unterschrift , Stempel**

**6.3. Einverständniserklärung des Direktors der Klinik, Abteilung, des Institutes**<br>Ich bin durch den Projektleiter über die in meiner Einrichtung geplanten Studie informiert worden und mit der Durchführung einverstanden. (Unterschrift, Name in Druckbuchstaben und Stempel)
**Name, Datum, Unterschrift, Stempel**

**6.4. ggf. Erklärung zur Vollmacht**, falls der Antrag (auch) für Dritte eingereicht wird (beispielsweise durch Studienzentralen, Sponsoren, Auftragsforschungsunternehmen/CROs u.ä.):<br><br>Ich versichere, für die Beteiligten / zu beratenden Ärztinnen und Ärzte zur Einreichung bei der Ethik-Kommission beauftragt und bevollmächtigt worden zu sein. Ich weiß, dass die Ethik-Kommission die Vorlage von Vollmachten verlangen kann.<br>Ich verpflichte mich, jedes Beratungsergebnis allen Beteiligten zur Kenntnis zu geben.
**Name, Datum, Unterschrift**

7. Erklärung zu Gebühren:

Für die Tätigkeit der Ethik-Kommission erhebt die Ärztekammer Westfalen-Lippe Gebühren nach Maßgabe der Verwaltungsgebührenordnung in der jeweils geltenden Fassung. Gebührenschuldner ist der Antragsteller, d.h. bei einer Beratung nach § 15 Abs. 1 Berufsordnung ÄKWL im Regelfall der für das Forschungsvorhaben verantwortliche Arzt.<br><br>Wenn hier nichts Abweichendes angegeben ist, ergeht der Gebührenbescheid an den unter Ziffer 1.1 genannten verantwortlichen Projektleiter.<br><br>Bitte stellen Sie sicher, dass Feld 2.7 zur Finanzierung ausgefüllt ist.

**Abweichender Rechnungsempfänger:**	☒ JA ☐ NEIN
Der Gebührenbescheid soll **ausgestellt** werden auf:<br><br>Firma<br>Abteilung<br>Ansprechpartner<br>Anschrift<br><br>ggf. Zusatzangaben	**Evang. Klinikum Bethel (EvKB)**<br>**Universitätsklinik für Psychiatrie und Psychotherapie, Forschungsabteilung**<br>**Prof. Dr. Martin Driessen (Chefarzt)**<br>**Remterweg 69-71, 33617 Bielefeld**
Der so adressierte Gebührenbescheid soll **gesendet** werden an:<br><br>Firma<br>Abteilung<br>Ansprechpartner<br>Anschrift<br><br>ggf. Zusatzangaben	**Evang. Klinikum Bethel (EvKB)**<br>**Universitätsklinik für Psychiatrie und Psychotherapie, Forschungsabteilung**<br>**Prof. Dr. Martin Driessen (Chefarzt)**<br>**Remterweg 69-71, 33617 Bielefeld**

6. Abschließende Erklärungen

6.1 Titel des Vorhabens hier wiederholen

Start in Ausbildung und Beruf – StAB

6.2 Unterschrift des/ der Antragsteller(in/nen), Stempel der Einrichtung
(bei mehreren verantwortlichen Projektbeteiligten Unterschriften aller Beteiligten)

**Name, Datum, Unterschrift, Stempel**<br>Lorenz Dehn 14.01.22<br>EVANGELISCHES KLINIKUM Bethel gGmbH<br>Klinik für Psychiatrie u. Psychotherapie<br>Bethel · Haus Gilead IV<br>Forschungsabteilung<br>Remterweg 69-71 · 33617 Bielefeld<br>Tel. 0521 772-78510 · Fax 0521 772-78511<br>evkb.de	**Name, Datum, Unterschrift , Stempel**
**Name, Datum, Unterschrift , Stempel**	**Name, Datum, Unterschrift , Stempel**

6.3. Einverständniserklärung des Direktors der Klinik, Abteilung, des Institutes
Ich bin durch den Projektleiter über die in meiner Einrichtung geplanten Studie informiert worden und mit der Durchführung einverstanden. (Unterschrift, Name in Druckbuchstaben und Stempel)

Name, Datum, Unterschrift, Stempel

Thomas Beblo 14.01.2022

EVANGELISCHES KLINIKUM Bethel gGmbH
Klinik für Psychiatrie u. Psychotherapie
Bethel · Haus Gilead IV
Forschungsabteilung
Ltg.: Prof. Dr. Thomas Beblo, Dipl.-Psych.
Remterweg 69-71 · 33617 Bielefeld
Tel. 0521 772-78512 · [illegible]
thomas.beblo@evkb.de
evkb.de

6.4. ggf. Erklärung zur Vollmacht, falls der Antrag (auch) für Dritte eingereicht wird (beispielsweise durch Studienzentralen, Sponsoren, Auftragsforschungsunternehmen/CROs u.ä.):

Ich versichere, für die Beteiligten / zu beratenden Ärztinnen und Ärzte zur Einreichung bei der Ethik-Kommission beauftragt und bevollmächtigt worden zu sein. Ich weiß, dass die Ethik-Kommission die Vorlage von Vollmachten verlangen kann.
Ich verpflichte mich, jedes Beratungsergebnis allen Beteiligten zur Kenntnis zu geben.

Name, Datum, Unterschrift

Gartenstraße 210 – 214
48147 Münster, Germany
Tel.: +49 (0)251 929 2460
Fax: +49 (0)251 929 2478
E-Mail: ethik-kommission@aekwl.de
www.ethik-kommission.uni-muenster.de

Ethik-Kommission Münster · Gartenstraße 210 – 214 · 48147 Münster

Herrn
Dr. rer. Nat. Lorenz Dehn, M.Sc.-Psych.
Evangelisches Klinikum Bethel (EvKB)
Universitätsklinikum OWL der Universität
Bielefeld, Universitätsklinik für Psychiatrie &
Psychotherapie
Remterweg 69/74
33617 Bielefeld

<u>nur per E-Mail:</u>
Lorenz.Dehn@evkb.de

2. März 2022 CS

**Aktenzeichen (bitte immer mit angeben):**	**2022-037-f-S**
Titel des Forschungsvorhabens:	Start in Ausbildung und Beruf (StAB)
Hier:	01_Neuantrag als zuständige EK, Eingang 17.01.2022

Sehr geehrter Herr Dr. Dehn,

für das oben genannte Forschungsvorhaben haben Sie die Beratung durch die Ethik-Kommission beantragt. Die Ethik-Kommission hat über Ihren Antrag beraten, dabei auch ergänzende/überarbeitete Unterlagen berücksichtigt und beschlossen:

Die Ethik-Kommission hat keine grundsätzlichen Bedenken ethischer oder rechtlicher Art gegen die Durchführung des Forschungsvorhabens.

Die vorliegende Einschätzung gilt für das Forschungsvorhaben, wie es sich auf Grundlage der in Anhang 1 genannten Unterlagen darstellt.

Für die Entscheidung der Ethik-Kommission erhebt die Ärztekammer Westfalen-Lippe Gebühren nach Maßgabe ihrer Verwaltungsgebührenordnung. Über die Gebühren ergeht ein gesonderter Bescheid.

<u>Allgemeine Hinweise:</u>

Mit der vorliegenden Stellungnahme berät die Ethik-Kommission die der Ärztekammer Westfalen-Lippe angehörenden Ärztinnen und Ärzte zu den mit dem Forschungsvorhaben verbundenen berufsethischen und berufsrechtlichen Fragen gemäß § 15 Absatz 1 Berufsordnung ÄKWL.
Falls Sie den Antrag im Namen weiterer ärztlicher Beteiligter gestellt haben, sind Sie verpflichtet, dieses Beratungsergebnis allen teilnehmenden Ärztinnen und Ärzten zur Kenntnis zu geben.

Die Einschätzung der Ethik-Kommission ist als ergebnisoffene Beratung für den Antragsteller nicht bindend. Unabhängig von der vorliegenden Stellungnahme verbleibt die medizinische, ethische und rechtliche Verantwortung für die Durchführung des Forschungsvorhabens bei dessen Leiter, Auftraggeber und bei allen an dem Vorhaben beteiligten Ärztinnen und Ärzten.

An der Beratung und Beschlussfassung haben die in Anhang 2 aufgeführten Mitglieder der Ethik-Kommission teilgenommen.

Die Ethik-Kommission der Ärztekammer Westfalen-Lippe und der Westfälischen Wilhelms-Universität Münster ist organisiert und arbeitet gemäß den gesetzlichen Bestimmungen und den GCP-Leitlinien der ICH.

Mit freundlichen Grüßen

Ch. Frantz

Prof. Dr. phil. Christiane Frantz
Stellv. Vorsitzende der Ethik-Kommission

Anhang 1

Folgende Unterlagen haben der Ethik-Kommission vorgelegen:
Bei mehreren Versionen eines Dokuments bezieht sich die Bewertung auf die neueste:

Eingang	Datierung	Anlage
17.01.2022	17.01.2022	Erstantrag Start in Ausbildung und Beruf (StAB)
17.01.2022	17.01.2022	StAB_1_EK_Anschreiben
17.01.2022	17.01.2022	StAB_2_EK_Antrag_komplett
17.01.2022	17.01.2022	StAB_3_EK_Anlage1_Aufkl_Einwill_Datensch_Teilnehmende
17.01.2022	17.01.2022	StAB_3_EK_Anlage2_Aufkl_Einwill_Datensch_Sorgeberecht
17.01.2022	17.01.2022	StAB_3_EK_Anlage4_Zuwendungsbescheid_Jobcenter-EvKB
17.01.2022	17.01.2022	StAB_3_EK_Anlagen3_Fragebögen_Entwurf
17.01.2022	17.01.2022	StAB_4_EK_CV_Projektleiter_Dehn_2022
24.02.2022	24.02.2022	AW Ethikantrag Start in Ausbildung und Beruf (StAB) unser Aktenzeichen 2022-037-f-S
24.02.2022	24.02.2022	StAB_EKMS_Formular_nicht-ärztliche-Forschungsvorhaben_V2-22-02-23

Anhang 2

An der Beratung und Beschlussfassung haben folgende Mitglieder der Ethik-Kommission teilgenommen:

Ärztinnen und Ärzte
Prof. Dr. med. Dirk Föll, Münster
Univ.-Prof. Dr. med. Karin Hengst, Münster
Prof. Dr. med. Heinrich Schulze Mönking, Telgte
PD Dr. med. Dirk Wähnert, Bielefeld

Zahnärztinnen und Zahnärzte
PD Dr. med. dent. Anne Wolowski, Münster

Apothekerinnen und Apotheker
Hans-Theo Fortmeier, Havixbeck

Personen mit Erfahrung auf dem Gebiet der Versuchsplanung und Statistik
Dr. rer. nat. Joachim Gerß Dipl. -Stat., Münster

Personen mit Befähigung zum Richteramt / Juristen
Dr. jur. Sascha Rolf Lüder, Düsseldorf

Personen mit wissenschaftlicher Erfahrung auf dem Gebiet der Ethik in der Medizin
Pfarrer Frank Neumann, Münster

Personen aus dem Bereich der Patientenvertretungen; Laien
Annette Hünefeld Dipl. Päd., Bonn

Weitere Fachrichtungen
Michael Finke Dipl.-Päd., MAE, Osnabrück
Prof. Dr. phil. Christiane Frantz, Münster
